# Risk prediction model for early detection of urinary tract infection in a hospital setting in Australia

**DOI:** 10.1177/18333583251363725

**Published:** 2025-08-21

**Authors:** Angela Jacques, Elizabeth Lloyd, Syed Aqif Muhktar, Pammy Yeoh, Brendon Mcmullen, Aron Chakera, Jim Codde

**Affiliations:** 1 The Institute for Health Research, University of Notre Dame Australia, Australia; 2 Sir Charles Gairdner Hospital, Australia; 3 North Metropolitan Health Service, Australia

**Keywords:** urinary tract infections, risk assessment, clinical decision rules, models, statistical, patient safety, electronic health records, health information management, predictive modelling, decision support, infection prevention

## Abstract

**Background:**

Hospital-acquired complications have detrimental effects on patient outcomes and recovery, with increased morbidity and mortality burdens, and hospital efficiency. The Australian Commission on Safety and Quality in Healthcare has identified 16 high-priority complications, including healthcare-associated infections, as potential targets of clinical risk mitigation strategies. Within the North Metropolitan Health Service in Western Australia, the prevalence of urinary tract infections (UTIs) was recognised as one of the most ubiquitous hospital-acquired complications and thus, there was desire to find new and innovative ways to enhance the existing infection prevention and control practices.

**Objective:**

To develop a risk prediction model for early identification of inpatients at risk of acquiring a UTI, to support clinical processes to facilitate targeted intervention strategies.

**Method:**

Prognostic modelling techniques were employed using retrospective hospital separation data encompassing patient and local health service factors.

**Results:**

The risk prediction model, developed from approximately 350 variables, used just 9 factors: 2 patient characteristics (age, gender), 4 clinical factors (paraplegia, dementia, prostate hyperplasia, neurosurgeon care), and 3 process measures (hospital stay duration, long theatre time, intensive care unit stay). It predicted UTI risk with 91% sensitivity, 86% specificity, and 95% discrimination (area under the curve). Real-time use in ward settings suggested it could help reduce hospital-acquired urinary tract infections (HAUTIs).

**Conclusion:**

Predictive modelling techniques can identify patients at risk of developing a HAUTI with high sensitivity and specificity. The resulting model can be used as a real-time clinical decision-making tool to guide proactive interventions and help reduce the prevalence of UTIs among hospital inpatients.

**Implications for health information management practice:**

The development and successful validation of a real-time predictive model for HAUTIs demonstrates how health information managers can leverage routinely collected data to support proactive clinical risk mitigation. Integrating such models into electronic health record systems can enhance patient safety, improve clinical workflows, and inform targeted infection control interventions across hospital settings.

## Introduction

Hospital-acquired complications (HACs) present significant challenges to healthcare systems, increasing patient morbidity, extending hospital stays and driving up healthcare costs (Hensley and Monson, 2015; Slawomirski and Klazinga, 2022). Among HACs, hospital-acquired urinary tract infections (HAUTIs) are both prevalent and preventable, accounting for approximately 20–40% of all healthcare-associated infections (Medina-Polo et al., 2021), particularly affecting patients with indwelling catheters or prolonged hospital stays (Meddings et al., 2019). HAUTIs not only contribute to patient discomfort and higher antibiotic use but also add a substantial financial burden to hospitals due to prolonged admissions and additional treatment requirements (Australian Commission on Safety and Quality in Health Care [ACSQH], 2018a; Medina-Polo et al., 2021), and led to calls for improved surveillance and prevention efforts for hospital patients at risk for urinary tract infections (UTIs), particularly in the non-intensive care unit (ICU) setting (Kelly et al., 2024). Without timely intervention, these patients also face an increased risk of sepsis and mortality (Sabih and Leslie, 2024). A recent systematic review reported that quality improvement interventions were associated with large declines in infection rates but as net costs to hospitals varied greatly, future research should examine specific practices associated with clinical effectiveness to improve cost savings (McCleskey et al., 2022).

Predictive models have been recognised as valuable tools for assessing individual patient risk and supporting clinical decision in hospital settings such as emergency departments (ED) for radiography requirements in acute knee injury presentations (Stiell et al., 1995); ICU for mortality risk (Labarére et al., 2014); oncology settings to predict recurrence-free survival after surgical resection (Gold et al., 2009); warning systems for hospital inpatient UTI risk (Møller et al., 2021). HAUTIs contribute significantly to hospital costs globally, with estimates ranging from A$8100 to A$36,400 per case (Agency for Healthcare Research and Quality, 2017). In Australia, hospitals also have an additional financial incentive to reduce HACs under the Independent Health and Aged Care Pricing Authority (IHACPA) framework (Webster et al., 2023). The IHACPA’s risk adjustment model complexity group estimates expected HAC levels based on patient complexity so that hospitals with higher-than-expected HAC rates can face funding reductions (Independent Health and Aged Care Pricing Authority [IHACPA], 2023). Despite these financial incentives, and the availability of a range of prevention strategies (Bausch et al., 2024; Rosenthal et al., 2025) a standardised approach to predicting and preventing HAUTIs remains lacking.

By implementing predictive analytics, it may be possible for hospitals to proactively reduce infection rates, improve patient safety and optimise healthcare resources. The aim of this study was to develop a predictive framework that identifies and validates key prognostic markers associated with early HAUTI onset, laying the foundation for a robust real-time clinical tool to improve patient safety and optimise healthcare resource allocation.

## Method

As part of the range of services offered by the North Metropolitan Health Service (NMHS), Western Australia, its tertiary hospital provides adult emergency and tertiary – quaternary acute care, while its associated specialist (secondary) hospital provides aged care and rehabilitation; same-day and elective surgery; and obstetrics and gynaecology services. These hospitals treat approximately 110,000 and 15,000 inpatients annually, respectively.

This retrospective cohort study utilised multiday inpatient separations data from these two sites that occurred between 1 July 2020 and 30 July 2022 (25 months). A multiday patient is defined as a patient who was being admitted into a hospital for at least one overnight stay. Same day cases were excluded from the study as it is not possible to determine whether a hospital acquired infection, such as UTIs, was a preexisting condition. The inpatient information was linked with ED data for those patients admitted following an ED presentation. Data were extracted from the NMHS data warehouse and linked by the Business Intelligence Unit.

### Data variables

To develop a predictive model for identifying patients at risk of UTI, all relevant data from the hospital’s inpatient and ED datasets were used (See Appendix 1). This comprehensive dataset included previously described UTI risk factors (Girard et al., 2017; IHACPA, 2018). During pre-processing, any duplicate patient records from the same episode of care were removed, and data cleaning consisted of checking for implausible values, and cross-checking key variables for accuracy. For efficiency, some variables were recoded into fewer categories or into categorical format if indicated in original distributions. The primary outcome was a clinically confirmed hospital-acquired UTI during admission (binary: yes/no), identified through post-discharge coding using ICD-10-AM code N39.0 and a date of onset flag indicating the condition developed during the hospital stay. After data cleaning, the dataset was split into a ‘model development’ set (1 July 2020 to 30 April 2022, 22 months), and an ‘external validation’ set (1 May 2022 to 31 July 2022, 3 months) for model validation.

### Sample size and power

A sample size calculation, using Stata user-written module *pmsampsise* (Ensor, 2018), indicated a sample of 7670 records that were required for development of a prediction model with an outcome prevalence = 0.7%, *C*-statistic = 0.95 and less than or equal to 30 model parameters (Pate et al., 2023; Riley et al., 2020).

### Statistical methods

Data analysis was performed using Stata version 18.0 (StataCorp, College Station, TX, USA). The Transparent Reporting of a Multivariable Prediction Model for Individual Prognosis or Diagnosis (TRIPOD) guidelines were followed (Collins et al., 2014). Significance was set at alpha = 0.05 during the data reduction phases, after which confidence intervals were used.

### Predictive model development

The model development dataset was used to develop predictive models as follows:

1. Variable selection and initial analysis: The initial data reduction phase consisted of univariate tests to assess the relationship between available variables and the presence of UTIs. These included *t*-tests or Mann–Whitney *U* tests for continuous variables and Chi-squared or Fisher’s exact tests for categorical factors. Data were summarised using means and standard deviations or medians and interquartile ranges for continuous variables, while categorical variables were presented as frequency distributions. Variables that showed no statistically significant differences between UTI groups or had very low frequencies (less than five) were excluded from further analysis.
2. Exploratory logistic regression and data reduction: A secondary screening process involved exploratory logistic regression to evaluate the strength and direction of associations between variables and UTIs. Variables were assessed both individually (univariately) and after adjusting for length of stay (LOS), age at admission, and female gender – factors known to be associated with UTI risk. Covariate interactions were also examined to assess their impact on UTI prediction.
3. Predictor selection using Least Absolute Shrinkage and Selection Operator (LASSO) regression: To refine the model, LASSO penalised regression was used to select a final subset of predictors from the variables identified during data screening (Tibshirani, 2018). This technique enhances risk prediction accuracy and prevents model overfitting, particularly when the number of events is low relative to the number of predictors (Pavlou et al., 2015).

### Refining the model with fractional polynomial terms

To optimise the model, a fractional polynomial term for LOS was incorporated to better capture the relationship between this continuous predictor and the outcome. This flexible approach enhances predictive accuracy by accounting for complex and non-linear relationships (Sauerbrei et al., 2020). By adapting to various data patterns, this method improves performance and provides a more nuanced understanding of how continuous variables influence UTI risk. In the final predictive model, LOS was represented as [ln(LOS) + square root(LOS)]; a −2, 0 fractional polynomial.

### Model evaluation and performance assessment

Model fit was evaluated using the Hosmer–Lemeshow goodness-of-fit test (Nattino et al., 2020) and the Akaike information criterion (Bozdogan, 1987; Gold et al., 2009).

Performance was assessed using:

- Concordance (c) index, measured by the area under the receiver operating characteristic curve (ROC AUC; Hajian-Tilaki, 2013; Longato et al., 2020).
- Sensitivity and specificity metrics.
- Deviance (−2 log-likelihood) to measure model fit.
- Brier scores to assess overall prediction accuracy (Steyerberg et al., 2010).

### Validity and reliability

Internal validation (Hemingway et al., 2013) was conducted to assess predictive accuracy using discrimination and calibration measures.

- Discrimination, which evaluates how effectively the model differentiates between outcomes, was assessed using 10-fold cross-validation and bootstrapping with replacement to generate confidence intervals. To avoid overfitting and improve predictive performance estimation, split-sample validation was not used (Steyerberg and Vergouwe, 2014).
- Calibration, which examines the accuracy of predicted probabilities, was assessed visually using calibration belt plots (Fenlon et al., 2018; Finazzi et al., 2011; Nattino et al., 2014, 2017). Additionally, calibration test statistics and Hosmer–Lemeshow tests (Kramer and Zimmerman, 2007) were used to evaluate deviations from perfect calibration.
- Discrimination plots were used to visually demonstrate how well the model distinguished between outcomes (Steyerberg et al., 2010).

### External statistical validation

External validation (Moons et al., 2012) was performed to assess the model’s generalisability to different data using the external validation set comprising hospital separation records occurring after the extraction period of the model development set.

### Final model and risk score calculation

Once the penalised logistic regression model was finalised and validated, the linear predictor (log odds) was calculated as the coefficient-weighted sum of included variable values:

$$\log\frac{p(\mathrm{UTI})}{1-p(\mathrm{UTI})}=\beta_{0}+\beta_{1}X_{1}+\ldots +\beta_{k}X_{k}=X\beta$$

From this, the predicted probability of a hospital-acquired UTI was derived using:

$$p\left(\mathrm{UTI}\right)=\frac{\exp(X\beta )}{1+\exp(X\beta )}$$

This probability was then converted into a percentage risk score using:

p(UTI)×100

Sullivan et al. (2004).

A threshold cut-point maximising discrimination for UTI was identified using Youden’s Index (Schisterman et al., 2008). The percentage risk score was compared multiplicatively and additively to the identified cut-point to obtain relative and absolute risk of individual percentage risk score compared to the cut-point.

Relativerisk=percentageriskscore/cut-point

$$\begin{array}{l} \mathrm{Predicted}\;\mathrm{risk}\;\mathrm{as}\;a\;\mathrm{difference}\;\mathrm{from}\;\mathrm{cut}-\mathrm{point}\\ =\mathrm{percentage}\;\mathrm{risk}\;\mathrm{score}-\mathrm{cut}-\mathrm{point} \end{array}$$

### Impact study

To evaluate the prediction model’s practical utility in a clinical setting, a prospective study was conducted in two neurosurgery wards from 1 March to 30 May, 2023. The study’s aim was to assess the model’s operational acceptability and its impact on HAUTI rates.

The predictive model was integrated into a Microsoft Power BI dashboard electronically linked to the hospital’s inpatient data systems, which provided most of the required variables. Nursing staff however were required to manually indicate, once-off, whether patient’s had paraplegia, dementia, and/or prostate issues. The dashboard calculated each patient’s daily UTI risk. Verbal feedback from staff was recorded throughout the study. Upon completion, patient discharge data were extracted for analysis, and the HAUTI incidence and rate per 1000 bed days calculated. These results were then compared to historical data from the same wards during the equivalent period in the previous year (1 March to 30 May, 2022). Generalised linear regression models were employed to determine the pre–post risk ratio.

### Ethics

The intention to publish methods and results contained in this document was endorsed by the hospital Human Research Ethics Committee (HREC). Since this project was classified as a quality improvement initiative, a full ethics review was not required. However, it was formally acknowledged that authors had sought review from the HREC, and this activity aligns with the principles of the National Statement on Ethical Conduct in Human Research (Quality Improvement/Audit project 47440).

## Results

The *model development dataset* consisted of 65,945 hospital separations of which 57,226 records (86.8%) were from the tertiary hospital with the remaining 8719 (13.2%) from the specialist hospital. This included 3149 (4.8%) cases that recorded a HAC, with 430 these being due to HAUTI (0.7%). The majority of the HAUTI cases (79.2%) were patients who were otherwise fit and healthy with no comorbidities at the time of admission and were classified as ‘low complexity’ as defined in IHACPA’s Risk Adjustment Model Complexity Group (IHAPCA, 2023). Of the remaining records, 10.3% were classified as ‘moderate complexity’ (at the time of admission, the patients’ medical history included hypertension and type 2 diabetes managed with oral medication) and 10.5% as ‘high complexity’ (at the time of admission, the patients’ medical history included dementia, cirrhosis of the liver, chronic renal failure, chronic obstructive pulmonary disease and type 2 diabetes managed with insulin). The initial data exploration phase, utilising tests of equality to explore differences between UTI and non-UTI groups, reduced the number of variables in the ‘model development’ set from 350 to 70. These were then examined for significant associations with the UTI outcome, using univariate and adjusted logistic regression models, resulting in 37 candidate variables for the LASSO regression (Table 1).

**Table 1.** Exploratory logistic regression modelling: statistically significant variables.

	Univariate OR			Adjusted OR		
	OR	95% CI	*p*	OR	95% CI	*p*
Gender: Female	1.52	1.25, 1.84	<0.001	1.82	1.48, 2.24	<0.001
Age categories: (reference <35)						
35–49	4.56	2.48, 8.38	<0.001	1.80	0.87, 3.73	0.114
50–64	6.68	3.71, 12.00	<0.001	3.26	1.73, 6.15	<0.001
65–79	7.80	4.33, 14.06	<0.001	5.93	3.24, 10.84	<0.001
⩾80	2.42	1.22, 4.77	0.011	6.86	3.75, 12.55	<0.001
Chronic diseases (any)	7.18	3.94, 13.07	<0.001	2.77	1.49, 5.14	0.001
Comorbidities						
Cancer	1.57	1.23, 2.01	<0.001	1.48	1.15, 1.92	0.002
Paraplegia	7.00	5.41, 9.07	<0.001	3.33	2.50, 4.44	<0.001
Peripheral vascular	2.95	1.72, 5.04	<0.001	2.40	1.36, 4.24	0.003
Prostate issues	3.72	1.97, 7.01	<0.001	3.85	1.92, 7.72	<0.001
Dementia	6.45	5.28, 7.88	<0.001	2.64	2.09, 3.34	<0.001
CVA	5.04	4.03, 6.30	<0.001	2.91	2.29, 3.71	<0.001
Major diagnostic category: nervous system	3.39	2.48, 4.63	<0.001	2.97	2.14, 4.13	<0.001
Transfer admission	5.22	4.29, 6.36	<0.001	2.21	1.76, 2.78	<0.001
ED arrival mode: ambulance, air ambulance (Royal Flying Doctor Service)	2.30	1.65, 3.22	<0.001	1.71	1.18, 2.49	0.005
ED departure destination = theatre	10.54	5.91, 18.80	<0.001	9.10	4.81, 17.23	<0.001
Highly specialised procedure	4.30	3.42, 5.41	<0.001	4.16	3.23, 5.36	<0.001
ICU indicator	6.84	5.46, 8.58	<0.001	4.61	3.57, 5.96	<0.001
Single-room indicator	2.24	1.85, 2.71	<0.001	1.75	1.43, 2.15	<0.001
Catheter procedure	2.09	1.04, 4.23	0.040	2.21	1.06, 4.59	0.034
Surgery indicator	1.64	1.36, 1.98	<0.001	2.33	1.89, 2.87	<0.001
Long theatre time (>2 hours)	2.97	2.44, 3.63	<0.001	3.22	2.59, 3.99	<0.001
In-hospital fall	7.39	5.46, 10.00	<0.001	1.71	1.19, 2.46	0.004
Continuous mechanical ventilatory support	9.15	6.73, 12.45	<0.001	4.49	3.11, 6.50	<0.001
Number ward movements >2	3.11	2.45, 3.94	<0.001	2.44	1.88, 3.16	<0.001
Presence of another HAC	101.47	82.80, 124.35	<0.001	49.19	38.79, 62.38	<0.001
LOS (days)	1.06	1.06, 1.07	<0.001	1.06	1.06, 1.07	<0.001
Separated care type: rehabilitation	6.45	5.13, 8.10	<0.001	1.52	1.15, 2.00	0.003
Separated team						
Geriatric acute and rehabilitation medicine	7.98	4.91, 12.95	<0.001	3.73	2.24, 6.24	<0.001
Geriatric evaluation and management	5.83	3.74, 9.10	<0.001	3.70	2.32, 5.90	<0.001
General medical	9.40	5.47, 16.17	<0.001	4.20	2.32, 7.57	<0.001
Neurosurgery	13.77	10.07, 18.82	<0.001	14.16	9.98, 20.10	<0.001
Rehabilitation	5.40	3.36, 8.68	<0.001	6.03	3.68, 9.90	<0.001
Separated to						
ICU	9.90	7.26, 13.51	<0.001	5.75	4.03, 8.21	<0.001
Neurosurgery	8.15	6.35, 10.48	<0.001	8.18	6.25, 10.69	<0.001
Gerontology	6.87	5.52, 8.54	<0.001	2.45	1.90, 3.16	<0.001
Diagnostic-related group: surgical	2.50	1.28, 4.88	0.007	2.31	1.17, 4.55	0.016

HAC: hospital-acquired complication; ICU: intensive care unit; ED: emergency department; CVA: cerebrovascular accident; LOS: length of stay; OR: odds ratio; CI: confidence interval.

### Outcomes of LASSO predictor regression

The 37 candidate variables were further reduced through an iterative LASSO regression process to 9 variables that yielded the best fitting predictive model. These were age, gender, indicators for presence of paraplegia, dementia, prostate hyperplasia, intensive care admission, neurosurgery as clinical specialty and operating theatre duration exceeding 2 hours and LOS.

### Internal and external model validation

**Figure 1.**
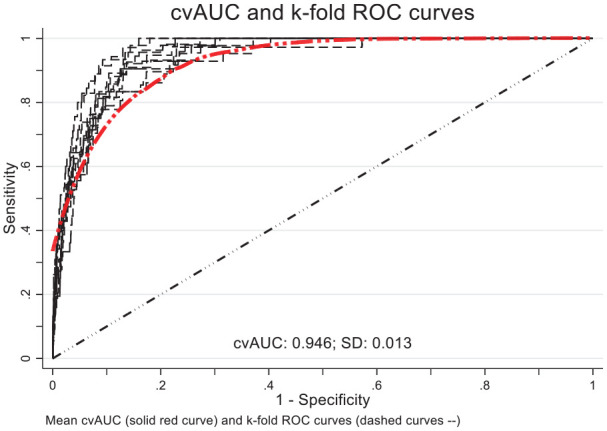
Ten-fold cross-validation ROC curves and AUC. Roc: receiver operating characteristic; AUC: area under the curve.

Performance validation test statistics for the final model are shown in Table 2. Data derived from the *model development dataset* achieved a deviance of 5.4%, with a Brier score of 0.006, indicating high accuracy and predictive precision. Eighty-six percent of HAUTI cases were correctly classified with an AUC of about 94.6% (Figure 1), indicating strong predictive accuracy.

**Table 2.** Model performance validation test statistics: Internal and external validation.

	Model development dataset	External validation dataset
	*n* = 65,938	*n* = 8316
AIC	3487	617
LL	−1732	−297
Brier score (tends to zero)	0.006	0.008
Cross-validated mean AUC	0.946	0.935
Bootstrapped AUC	0.957	0.935
Bootstrapped bias corrected AUC 95% CI	0.939, 0.953	0.912, 0.951
Sensitivity	90.93	94.67
Specificity	85.96	79.72
False positive rate (%)	14.0	23.0
False negative rate (%)	9.0	5.0
Correct classification	85.99	79.86
Calibration belt plot test statistic, deviance significance	*T* = 1.77, *p* = 0.099	*T* = 0.15, *p* = 0.699
Hosmer–Lemeshow goodness of fit test	χ^2^ = 6.19, *p* = 0.626	χ^2^ = 3.68, *p* = 0.885

AIC: Akaike information criterion; LL, log likelihood; AUC: area under the curve; CI: confidence interval.

**Figure 2.**
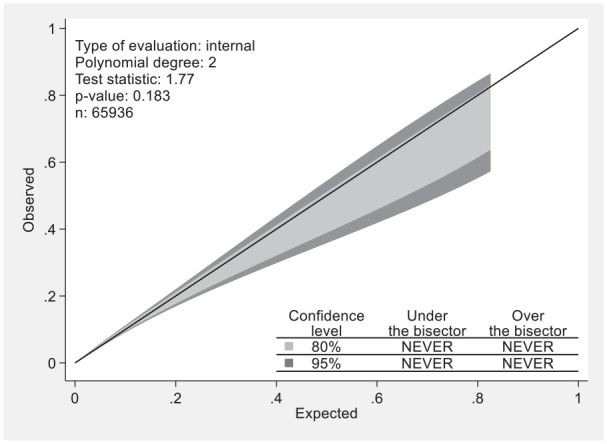
Calibration belt plot showing deviations from the line of perfect fit at 80% and 95% confidence levels.

**Figure 3.**
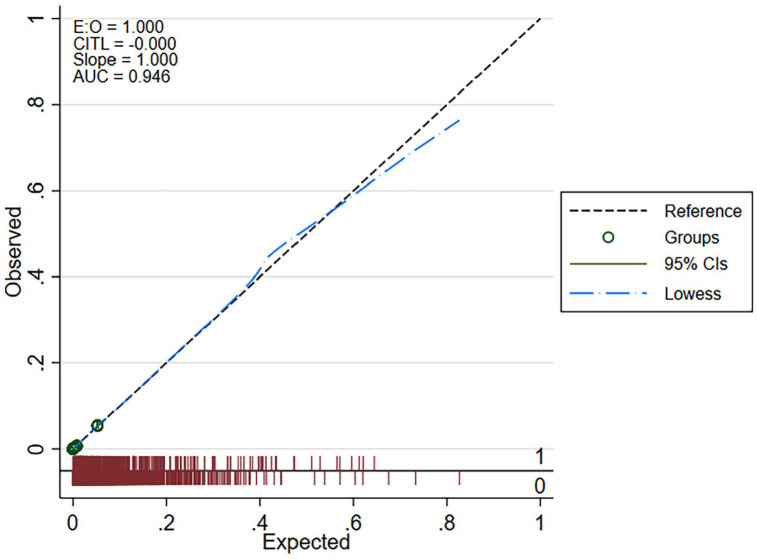
Discrimination plot.

The calibration belt plot shown in Figure 2 demonstrated excellent calibration, as both the 80% and 95% calibration belts bounded the bisector consistently throughout the full range of predicted probabilities, suggesting model prediction did not significantly deviate from the observed outcomes thus indicating reliable performance. The discrimination plot illustrated in Figure 3 demonstrates the model’s ability to accurately distinguish between patients with and without UTI. External validation of the developed model was undertaken using 8316 hospital separations that occurred immediately after the period used for the model development dataset. The model metrics for the *external validation dataset* indicated similar good performance.

### Final model equation

The equation coefficients in the validated final risk model were

$$\begin{array}{l} Y=\mathrm{intercept}+(\beta_{1}\chi_{1}+\beta_{2}\chi_{2}+\beta_{3}\chi_{3}+\beta_{4}\chi_{4}\\ +\beta_{5}\chi_{5}+\beta_{6}\chi_{6}+\beta_{7}\chi_{7}\\ +\beta_{8}\chi_{8}+\beta_{9}\chi_{9}+\beta_{10}\chi_{10}) \end{array}$$

Intercept=-9.723672+β1χ1=(0.0199277*Age)+β2χ2=(0.6208797*Femalegenderindicator)+β3χ3=(0.6221867*Paraplegiaindicator)+β4χ4=(0.4230232*Dementiaindicator)+β5χ5=(1.30041*Prostatehyperplasiaindicator)+β6χ6=(1.125256*Neurosurgeryindicator)+β7χ7=(0.3672426*ICUindicator)+β8χ8=(0.4456216*Longtheatretimeindicator)+β9χ9=(0.−14.94228*LOS^*2)+β10χ10=(1.389978*ln(LOS))

An individual’s probability of developing a UTI at that point in their hospital stay is estimated as follows:

ProbabilityofUTI=exp(Y)/[1+exp(Y)]

Percentagerisk=ProbabilityofUTI*100

$$\begin{array}{l} \mathrm{UTI}\;\mathrm{discrimination}\;\mathrm{risk}\;\mathrm{threshold}\\ \left(\mathrm{Youden}\;\mathrm{cut}\;\mathrm{point}\right)=1.5\% \end{array}$$

Relativerisk=Percentagerisk/1.5

Absoluterisk=Percentagerisk-1.5

The data in Table 3 demonstrate the changes in risk that can impact on the probability of a patient having a HAUTI due to age, gender, medical complexity, hospital processes (ICU, operating theatre) and duration of hospital stay. Classification of patients, based on stratified predicted relative risk, could be utilised in clinical decision-making to minimise patient’s risk of developing a UTI doing their hospital stay by targeting educational and preventative interventions.

**Table 3.** Predicted UTI risks for different hypothetical patients.

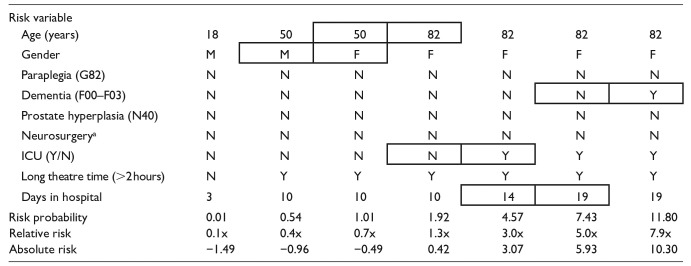

UTI: urinary tract infection; ICU: intensive care unit.

a Determined by clinical speciality of separation unit. Boxed cells are to highlight patient factor differences from previous- in order to demonstrate how 1 or 2 changes can impact patient risk quite substantially.

### Impact study results

The 3-month long impact study involved 209 and 185 neurosurgery patients in the pre-intervention and intervention periods, respectively. The incidence rate of HAUTI during the prospective trial was 1.6% compared to 5.3% in the earlier period (*p* = 0.053), with the adjusted relative risk (95% confidence interval) 0.34 (0.10, 1.22), adjusting for age, gender and days in hospital. Rate of HAUTI per 1000 bed days also decreased from 4.9% to 2.6% (*p* = 0.235). While the nursing staff found the dashboard useful to identify patients at risk of UTI, there was reluctance to manually enter data.

## Discussion

After analysing 350 data elements collected during a patient’s hospital care, the application of risk prediction modelling techniques identified 9 factors that demonstrated high accuracy and predictive precision in detecting those who developed HAUTIs. These factors consisted of two patient characteristics (age and gender), four clinical factors (paraplegia, dementia, prostate hyperplasia, under care of a neurosurgeon) and three process measures (duration of hospital stay, long theatre time and ICU stay). This model lends itself to readily being used to monitor a patient’s individual risk of developing a HAUTI during their admission as all the factors could be ascertained prior to discharge. Such a tool could help clinicians target specific patients at high risk of developing a HAUTIs by ensuring adherence of best practice guidelines (ACSQHC, 2018b, 2024; Centres for Disease Control National Healthcare Safety Network, 2025) and patient education in a more precise and resource effective way. This position was supported by the findings of our impact trial on the patient wards, where the incident rate of HAUTIs and rate per 1000 bed days were both reduced during the 3-month intervention period. Although this failed to reach statistical significance during the short intervention, a different outcome may have been achieved across a full year due to larger sample sizes.

Decision and risk prediction tools have been developed for a variety of clinical applications such as healthcare resource consumption and clinical outcome in cardiovascular patients (Takura et al., 2021), use of radiography in acute knee injuries (Stiell et al., 1995, 1996), prognosis of patients with non-specific neck pain (Schellingerhout et al., 2010), neurosurgical intervention (Orlando et al., 2017), recurrence-free survival after complete surgical resection of localised primary gastrointestinal stromal tumour (Gold et al., 2009), to name a few. In addition to these, others have reported predictive tools of patients at risk of acquiring UTIs while in ED (Flores et al., 2024) or during hospital admission (Jakobsen et al., 2024; Møller et al., 2021). While the level of interest in this area is encouraging, the level of performance, reliance of pathology test results or number of data variables required by the model indicates the need for other approaches, such as the model described in this article, which has demonstrated the potential for important patient benefits.

This study has demonstrated that the individualised prediction of HAUTI risk is feasible using routinely collected patient data at admission and during their period of hospital stay utilising a risk prediction model rather than reliance of logistic regression and machine language learning often used by other, as reported in a review by Luz et al. (2020) and other authors (Mukhtar et al., 2025; Sufriyana et al., 2025). While risk prediction models and statistical models share commonalities, they serve distinct purposes and have important differences between them (Shmueli, 2010). Statistical models encompass various techniques, including regression modelling, that can be used for prediction, explanation and hypothesis testing *within the sample of interest*. In contrast, risk prediction models are prognostic mathematical models designed to *estimate an individual’s probability of an event occurring* (Kent et al., 2020). In this study, logistic regression modelling was used to assist in data reduction of over 350 variables before LASSO modelling was used to identify the final candidate predictors in the risk model.

The predictive model showed excellent performance in a dual-site hospital setting, demonstrating strong discrimination and high accuracy. The model performed reliably in both the development and external validation datasets, indicating robust predictions for HAUTIs, supporting its clinical utility. It is also interesting to note that this predictive model only required nine factors to achieve the high-performance levels described above. Other recently released predictive models for UTIs have not achieved the same level of accuracy as in the current study, as measured by ROC curve analyse, despite utilising more predictive factors (Møller et al, 2021; Sufriyana et al., 2025) and relying on timely pathology results (Flores et al., 2024).

Like many researchers exploring predictive models for identifying patients at risk of HAUTIs, we believe this model can inform healthcare workers about which patients should receive targeted preventative strategies or early interventions during their hospital stay. That said, we are yet to find a publication that described the implementation of such a model in a clinical setting. In part, the answer may lie in the concluding comments of Luz et al. (2020) who, after reviewing 52 studies that utilised machine learning techniques designed to help in the management of infectious diseases, said that use of the applications seemed limited due to the failure to build trust in these new technologies, adequately explain and interpret the models, and lack of independent model validation and clinical studies.

Another challenge for widespread acceptability of predictive models is that the factors influencing HAUTI risk may vary between hospitals due to differences in the range of services they provide. As found in our study, the association between LOS in hospital and the development of HAUTIs has been reported by others (Jeon et al., 2012; Mitchell et al., 2016; Toh et al., 2017). Similarly, others have reported that certain procedures, such as spinal or neurosurgery, are associated with an increased risk of HAUTI (Pham et al., 2020; Tominaga et al., 2016), which we also noted as a key prognostic factor strongly linked to HAUTI, emphasising the need for tailored interventions addressing specific risk factors within different hospital specialties. This parallel finding may not be surprising as the tertiary hospital in our study houses several specialised centres including neurosurgery, oncology and organ transplant units. Hence, while our study clearly demonstrates that relatively simple statistical techniques can create highly accurate predictive models for identifying patients at risk of HAUTIs, the factors included in these models may need to be tailored to the specific case mix profile of each hospital. With longitudinal studies demonstrating that data-driven, evidence-based and outcome-oriented programmes can promote clinical engagement that results in sustained reduction in HACs (Li et al., 2024), there is strong imperative to for hospitals to use algorithms such as the one described in this article to enhance patient safety and potentially reduce unnecessary operating costs.

### Limitations

While electronic health records provided a rich source of data for analysis, a key limitation of our study was the exclusion of unknown (latent) patient factors and uncertainty regarding the method and timing of UTI diagnoses that were not available to us at the time of this study. The assumption that UTIs were diagnosed based on objective pathology results, without insight into the clinician’s diagnostic process, also presents a challenge about the veracity of the details captured in the patient’s coded discharge records. Additionally, inability to be certain whether the infection was hospital-acquired, or pre-existing could lead to an overestimation of the impact of LOS in the predictive model, as the proportion of LOS directly attributable to hospital-acquired UTIs remains unknown. Finally, as acknowledged in the Discussion, while the developed predictive model was shown to be highly accurate within the hospital setting where it was developed, further studies based on data obtained from a different time periods should be used to confirm this. Similarly, its applicability to other Western Australian or Australian hospitals with similar and different case-mix profiles would potentially increase the value of the model as described.

### Future directions

Future research should focus on applying these methods to develop risk prediction tools for other HACs and exploring the generalisability of the developed model across hospitals with diverse patient case mixes. This would help ensure that the model is applicable beyond the initial dataset and can be effectively used in different healthcare settings. The value of these tools needs to be more fully explored in a clinical setting than was possible in the current study. In an operational setting, such tools will also need to address user concerns about adding to their workload but system integration with real-time patient information systems should help overcome this issue and make the tool more widely available across the hospital.

## Conclusion

While all Australian hospitals understand the importance of reducing the levels of hospital acquired conditions in their patients, through the efforts of ACSQHC and the funding incentives built into IHACPA national funding model, hospitals themselves are keen participants to improve patient safety and minimise unnecessary operational costs. Implementing a more refined and precise risk assessment strategy for early identification of patients at high-risk of HAUTI could significantly enhance the effectiveness of timely mitigation measures. By leveraging advanced machine-learning algorithms and predictive modelling, healthcare systems can proactively target at-risk patients for personalised care plans and interventions, which can lead to improved patient outcomes, reduced hospital stays and better overall management of infections within healthcare settings.

## Appendix

**Appendix 1.** Extracted variables and associated ICD-10 codes.

ED factors	ICD-10 AM
ED arrival mode	
Ambulance, flying doctor ambulance	
Other	
Private transport	
ED triage	
Resuscitation	
Emergency	
Urgent	
Semi urgent	
Non-urgent	
ED bed access (minutes)	
ED departure destination = theatre^a^	
Admission shift	
Emergency admission	
External transfer into ED	
Transferred from medical/residential	
ED fast track code	
ED flu-like symptoms	
ED diagnosis category	
Alcohol/drug use and alcohol/drug-induced organic mental disorders	F10–F19
Burns	T20–T31
Diseases and disorders of the blood and blood forming organs and immunological disorder	D50–D89
Diseases and disorders of the circulatory system	I00–I99
Diseases and disorders of the digestive system	K00–K95
Diseases and disorders of the ear, nose, mouth and throat	H60–H99
Diseases and disorders of the eye	H00–H59
Diseases and disorders of the female reproductive system	N80–N98
Diseases and disorders of the hepatobiliary system and pancreas	K80–K87
Diseases and disorders of the kidney and urinary tract	N18, N20, N25, N30, N39
Diseases and disorders of the male reproductive system	N00–N99
Diseases and disorders of the musculoskeletal system and connective tissue	M00–M99
Diseases and disorders of the nervous system	G00–G99
Diseases and disorders of the respiratory system	J00–J99
Diseases and disorders of the skin, subcutaneous tissue and breast	L00–L99
Endocrine, nutritional and metabolic diseases and disorders	E00–E89
Factors influencing health status and other contacts with health services	Z00–Z99
Infectious and parasitic diseases	A00–B99
Injuries, poisoning and toxic effects of drugs	S00–T98
Mental diseases and disorders	F00–F09
Neoplastic disorders (haematological and solid neoplasms)	C00–D49
Pregnancy, childbirth and the puerperium	O00–O99
ED episode time (hours)^a^	
<4	
4–8	
8–12	
12+	
ED mental health attendance	
ED disposition (13 categories)	
ED departure destination (7 categories)	
ED referral source	
Appointment/clinic	
General practitioner	
Other	
Self	
Admission/separation variables	
Admission day (7 categories)	
Admission day^a^	
Monday–Friday	
Saturday–Sunday	
Admission shift^a^	
8 am–4 pm (day)	
4 pm–12 am (evening)	
12 am–8 am (night)	
Admission shift^a^	
6 am–6 pm	
6 pm–6 am	
Separated care type	
Separated division	
Acute services	
Baby and neonate	
Cancer, imaging and clinical	
Obstetrics	
SCGOPHCG corporate	
Specialty and ambulatory	
Surgical	
Separated care type: acute	
Separated team (28 categories)	
Separated unit (41 categories)	
Separated unit/Dr	
Gerontology	
Other	
Separated destination (6 categories)	
Separated to health service (5 categories)	
Separation professional	
Community health clinician	
Emergency department clinician	
General practitioner	
Hospital clinician (re-admission)	
Other	
Outpatient department clinician	
Specialist clinician	
Statistical admission/type change	
Separated to accommodation type (11 categories)	
Separated to ICU^a^	
Separated to rehabilitation^a^	
Diagnostic variables	
Alcohol/drug use and alcohol/drug-induced organic mental disorders	F10–F19
Burns	T20–T31
Diseases and disorders of the blood and blood-forming organs and immunological disorder	D50–D89
Diseases and disorders of the circulatory system	I00–I99
Diseases and disorders of the digestive system	K00–K95
Diseases and disorders of the ear, nose, mouth and throat	H60–H99
Diseases and disorders of the eye	H00–H59
Diseases and disorders of the female reproductive system	N80–N98
Diseases and disorders of the hepatobiliary system and pancreas	K80–K87
Diseases and disorders of the kidney and urinary tract	N18, N20, N25, N30, N39
Diseases and disorders of the male reproductive system	N00–N99
Diseases and disorders of the musculoskeletal system and connective tissue	M00–M99
Diseases and disorders of the nervous system	G00–G99
Diseases and disorders of the respiratory system	J00–J99
Diseases and disorders of the skin, subcutaneous tissue and breast	L00–L99
Endocrine, nutritional and metabolic diseases and disorders	E00–E89
Factors influencing health status and other contacts with health services	Z00–Z99
Infectious and parasitic diseases	A00–B99
Injuries, poisonings and toxic effects of drugs	S00–T98
Mental diseases and disorders	F00–F09
Neoplastic disorders (haematological and solid neoplasms)	C00–D49
Pregnancy, childbirth and the puerperium	O00–O99
Patient characteristics	
Age	
Gender	
Interpreter required	
Comorbidities (CCI)	
CVA	160–169
CHF	I50.0–I50.4
Cancer	C00–C97
Paraplegia	G82
Renal disease	N12, N17–N19, N25–N28
Dementia	F00–F03
Pulmonary heart disease, chronic obstructive pulmonary disease	I26–I28, J44
Acute myocardial	I21.9
Peripheral vascular disease	I70–I74
Patient type	
Admitted service	
Nursing home type	
Transfer admission	
Complexity indicator	
Public patient	
Patient classification (3 categories)	
Marital status: partner^a^	
Prisoner	
Patient socio-economic index (deciles and quintiles)^a^	
Patient residential region (5 categories)^a^	
Patient remoteness (3 categories within WA)^a^	
CCI	
Patient comorbidities from CCI	
Acute myocardial	I21.9
Peripheral vascular disease	I70–I74
Pulmonary heart disease, chronic obstructive pulmonary disease	I26–I28, J44
Tissue diseases	L00–L99
Peptic ulcer	K27
Liver disease and related conditions	K73–K76, C22–C24
Diabetes	E10–E14
Cancer	C00–C97
HIV	B20–B24
Any comorbidities^a^	
Patient UTI risk factors (clinically defined)	
Spinal nerve/disc/stenosis	M48
Urethritis	N34
Nephritis	N12
Dysuria	R30
Male genital inflammatory	N49
Inflammatory prostate	N41
Orchitis epididymitis	N41.0, N45.9
Flank pain	R10
Renal disease	N12, N17–N19, N25–N28
Fever	N50
Connective tissue disease	M35
Hereditary nephropathology	Z84.1
Renal tubular impairment	N25
Urinary catheter	Z46.6
Mental health indicator	F09–F12, F33, F41, F43
Prostate hyperplasia	N40
Dementia	F00–F03
Diabetes complications	E10–E14
Fever	R50.9
CVA	160–169
Chronic diseases indicator^a^	
Chronic diseases count^a^	
In-hospital factors		
LOS		
LOS ⩾9 days^a^	
Long LOS (LOS >30 days)^a^	
LOS: age >65^a^	
LOS ICU (days)	
Single room indicator	
Urinary catheter	Z46.6
Urine test indicator	
Surgery indicator	
Long theatre time (>2 hours)a	
In-hospital fall (non-HAC)^a^	
Falls age >65^a^	
Number ward movements	
Room type		
High dependency	
ICU	
Psychiatric	
Shared	
Single	
CVS	
CVS duration	
Acute/subacute	
Number specialty movements	
Episode hours	
Highly specialised procedure indicator	
Highly specialised procedure count	
Neurosurgery	
Other indicated HACs	
Pressure injury	L89
Fall (injurious)	S02, S06–S07, S72
Sepsis	A40–A41, U90
Multi-resistant organism infection	Z06
Prosthetics/implant infection	T82
Surgical site infection	T81.4
Pneumonia	A02
Central vascular catheter infection	T80.21
Gastro-intestinal	A09.9
Gastro-intestinal bleeding	K92.2
Other high impact	Z91.89
Post-surgical return to theatre	Z48.81
Respiratory infection	J06.9
Venous thromboembolism	I82.90
Renal failure	N19
Medications complications	T88.7
Delirium	F05.9
Incontinence	R32
Malnutrition	E43–E44
Cardiac	I00–I99
Perineal lacerations	O70
Other HAC indicator	Y84.9

CVA: cerebrovascular accident; CHF: congestive heart failure; CVS: continuous ventilatory support; HAC: hospital-acquired complication; UTI: urinary tract infection; CCI: Charlson comorbidity index; ICU: intensive care unit; ED: emergency department; LOS: length of stay.

a Recoded variable.
